# The Pathogenetic Effect of Natural and Bacterial Toxins on Atopic Dermatitis

**DOI:** 10.3390/toxins9010003

**Published:** 2016-12-23

**Authors:** Kyung-Duck Park, Sok Cheon Pak, Kwan-Kyu Park

**Affiliations:** 1 Department of Dermatology, College of Medicine, Catholic University of Daegu, 33, Duryugongwon-ro 17-gil, Nam-gu, Daegu 42472, Korea; gdpk1217@naver.com; 2 School of Biomedical Sciences, Charles Sturt University, Panorama Avenue, Bathurst NSW 2795, Australia; spak@csu.edu.au; 3 Department of Pathology, College of Medicine, Catholic University of Daegu, 33, Duryugongwon-ro 17-gil, Nam-gu, Daegu 42472, Korea

**Keywords:** atopic dermatitis, toxin, pathogenesis

## Abstract

Atopic dermatitis (AD) is a common allergic skin disease that is associated with chronic, recurrent eczematous and pruritic lesions at the flexural folds caused by interacting factors related to environmental and immune system changes. AD results in dry skin, and immunoglobulin E-mediated allergic reactions to foods and environmental allergens. While steroids and anti-histamines temporarily relieve the symptoms of AD, the possibility of side effects from pharmacological interventions remains. Despite intensive research, the underlying mechanisms for AD have not been clarified. A study of *Staphylococcus aureus (S. aureus)* established the role of its toxins in the pathogenesis of AD. Approximately 90% of patients with AD experience *S. aureus* colonization and up to 50%–60% of the colonizing *S. aureus* is toxin-producing. Any damage to the protective skin barrier allows for the entry of invading allergens and pathogens that further drive the pathogenesis of AD. Some natural toxins (or their components) that have therapeutic effects on AD have been studied. In addition, recent studies on inflammasomes as one component of the innate immune system have been carried out. Additionally, studies on the close relationship between the activation of inflammasomes and toxins in AD have been reported. This review highlights the literature that discusses the pathogenesis of AD, the role of toxins in AD, and the positive and negative effects of toxins on AD. Lastly, suggestions are made regarding the role of inflammasomes in AD.

## 1. Introduction

The chronic inflammatory skin disease, atopic dermatitis (AD), produces eczematous and pruritic lesions at the flexural folds due to interacting factors that are related to environmental and immune system changes [1]. AD is a chronic, recurrent form of skin inflammation, involving a disturbance in the epidermal-barrier that results in dry skin, and an immunoglobulin E-mediated allergic reaction to foods and environmental allergens [2]. Histamine derived from skin mast cells (MCs) has been reported as an important itch mediator in AD lesions [3,4,5]. Varying epidemiologic factors including nutrition, the number of siblings, urban settings, social status, or climatic aspects can impact on the risk of AD [6,7,8]. The incidence of AD has increased dramatically in industrialized countries over the past three decades [8]. Indeed, most progress in knowledge concerning the immunologic mechanisms of AD has been gained in recent decades [9]. Despite intensive research, the underlying mechanisms for AD have not yet been clarified.

A study of *Staphylococcus aureus (S. aureus)* established the role of the *S. aureus*-produced toxins, especially α-toxin and enterotoxins, in the pathophysiology of AD [10]. It was further found that compared with only 5%–30% of nonatopic individuals, approximately 90% of AD patients experience *S. aureus* colonization, and that up to 50%–60% of the colonizing *S. aureus* is toxin-producing [11,12]. A recent systematic review showed that 70% of AD patients had *S. aureus* colonization on their lesional skin, 39% colonization on their non-lesional skin, and 62% on their nose, and meta-regression analysis has demonstrated that the increased prevalence of *S. aureus* colonization is related to disease severity [13]. Any damage to the protective skin barrier allows for the entry of invading allergens and pathogens that further drive the pathogenesis of AD.

While steroids and anti-histamines temporarily relieve the symptoms of AD, the possibility of side effects from pharmacological interventions remains [14]. To solve such problems, current therapies and research have brought about an improvement in clinical symptoms by targeting the specific pathways involved in the pathogenesis of AD. Meanwhile, some natural toxins (or their components) that have therapeutic effects on AD have been discovered [15]. Some studies have reported on the relationship between inflammasome, one component of the innate immune system, and toxins in AD [16,17,18,19].

This review highlights the literature that discusses the pathogenesis of AD, the role of toxins in AD, and the positive and negative effects of toxins on AD. Lastly, suggestions are made on the role of inflammasomes in AD.

## 2. Pathogenesis of AD from an Immunologic Point of View

**Figure 1:**
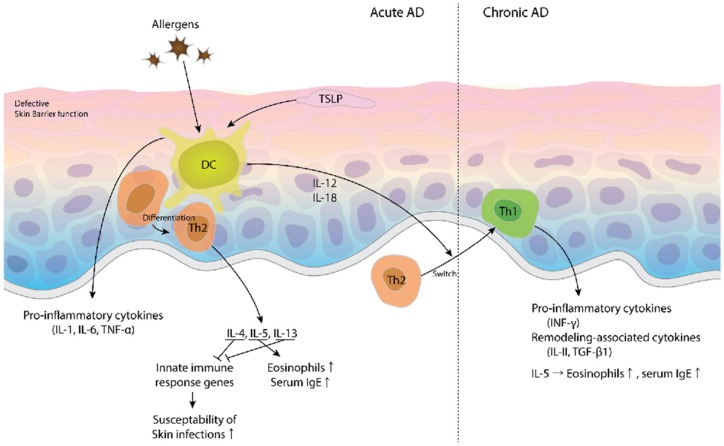
Acute and chronic phases of atopic dermatitis (AD) [1]. In acute AD, allergens induce differentiation of Th2 cells and secretion of the pro-inflammatory cytokines such as interleukin 1 (IL-1), IL-6, and tumor necrosis factor (TNF)-α from dendritic cells (DCs). Damaged keratinocyte-derived thymic stromal lymphopoietin (TSLP) also derives DCs for polarization toward Th2 cells [31]. IL-4 and IL-13 suppress the induction of innate immune response genes and increase susceptibility to skin infections. The production of the Th1-like cytokines IL-12 and IL-18 induces the switch from Th2 cells to Th1 cells, and thereby leads to the chronic phase of AD [32]. The secretion of remodeling-associated cytokines such as transforming growth factor (TGF)-β1 and IL-11 [33], eosinophil recruitment, and IL-5 production contribute to the maintenance of chronic AD.

AD is a chronic inflammatory, highly pruritic skin disease resulting from complex interactions between a defectively functioning skin barrier, systemic and local immunologic responses to microbial antigens and allergens, and susceptibility genes (Figure 1) [1]. Allergens in foods and in pollens, microbes, and house-dust-mite products penetrate the epidermis due to barrier dysfunction. Some molecules from pollens and foods drive dendritic cells to promote T helper cell 2 (Th2) polarization [20,21]. In a murine model, repeated epicutaneous exposures to ovalbumin induce ovalbumin-specific serum IgE, allergic asthma, and atopic dermatitis-like eczematous skin lesions [22]. AD lesions are observed when there is an increase in the infiltration of T cells, macrophages (Mфs), dendritic cell (DC) subtypes, eosinophils, MCs, as well as the secretion of various chemokines and cytokines [23,24]. Various mediators produced by cells in the skin attract T cells into the skin and cause chronic inflammation [16,25].

Patients with AD are prone to recurrent skin infections [26]. An early study suggested that decreased levels of antimicrobial peptides (AMPs) including human beta-defensin (hBD)-2, hBD-3, and cathelicidin, render AD skin more susceptible to skin infections [27]. In contrast, a recent study showed that although the levels of AMPs in AD lesions increased equivalently to those in healthy skin, this was still insufficient to defend against *S. aureus* infection. The possible reasons for this were attributed to the high levels of *S. aureus* colonization or to defects in AMP function [28]. The role of Toll-like receptors (TLRs) in innate immunity is important in recognizing pathogen-associated molecular patterns (PAMPs). In AD cases, the release of Th2 cytokines and the suppression of TLR expression are important factors for the increased incidence of skin infections [26]. Both the lesional skin and non-lesional skin of AD patients have intensive bacterial colonization, such as by *S. aureus*, which is known to stimulate TLR2 [29]. Recently, Song et al. confirmed that interactions between TLR2 activation and the upregulation of FcεRI expression occurred via the p38 pathway. This finding in patients with severe extrinsic AD might provide insight into how bacterial infection can aggravate the clinical symptoms of AD [30].

Several studies of acute AD have demonstrated the presence of Th2-like and cytokine-producing T cells that result in enhanced allergic skin inflammation. In the chronic phase of AD, the switching of Th2 cells into interferon (IFN)-γ-producing Th1-like cells occurs. The Th2 cytokines such as interleukin 4 (IL-4), IL-5, and IL-13 are reported to be predominant in the acute phase of AD, and in chronic AD lesions, an increase in IFN-γ, IL-12, IL-5, and granulocyte-macrophage colony-stimulating factor (GM-CSF) has been noted [34]. AD patients have increased numbers of T cells secreting IL-4 and IL-13 [35]. IL-4 and IL-13 mediate downstream signal transduction to suppress the innate immune response genes [27,36,37], thereby making AD patients more susceptible to skin infections with herpes simplex virus and *S. aureus* [38,39]. Therefore, Th2 cytokine-targeted therapies may provide new strategies in the treatment of AD patients [40].

Another important Th2 cytokine, IL-5, plays a key role in the proliferation, differentiation, and activation of eosinophils [41,42]. Both skin lesions and the peripheral blood of AD patients have elevated levels of IL-5 that significantly correlate with serum levels of IgE [43]. A murine model with the genetic deletion of IL-5 showed a reduction in skin eosinophilia and epidermal thickness after exposure to an allergen [44]. This finding suggests a blockade of IL-5 function is applicable to AD patients.

Thymic stromal lymphopoietin (TSLP), which activates DCs to promote Th2 cell differentiation [31], is released by damaged skin epithelial cells upon stimulation with allergens [45,46]. Overexpression of TSLP in the skin leads to increased serum levels of Th2 cytokines and IgE [45]. TSLP triggers the induction of IL-5 release and the recruitment of eosinophils [47], and the induction of the AD phenotype by TSLP in mice is dependent on T cells, but not on IL-4/IL-13 [48]. A recent study demonstrated that itch signaling was induced by TSLP via sensory neurons [49]. These findings suggest that TSLP has the potential to drive AD pathogenesis in a Th2-dependent and -independent manner while inducing the “itch cycle” in patients as well. A recent strong link has been demonstrated between TSLP and serum IL-31 and IL-33 levels, thus suggesting that TSLP is a new biomarker for AD [50]. Furthermore, a new Th2 cytokine, IL-31, has major pruritogenic potency for inflammatory mediators in AD [51], and serum levels of IL-31 correlate with disease severity in AD patients [52]. A recent piece of research has demonstrated that a monoclonal antibody targeting the IL-31 receptor A (IL-31RA) significantly relieved AD pruritus [53].

The maintenance of chronic AD requires the production of the Th1-like cytokines including IL-12 and IL-18, as well as of several remodeling-associated cytokines such as IL-11 and transforming growth factor (TGF)-β1 [33]. Further, the change from acute AD lesions to chronic AD lesions is accompanied by an influx of inflammatory cells and is associated with pro-inflammatory cytokines such as IL-6, tumor necrosis factor (TNF)-α, and IFN-γ [1,32]. The pleiotropic effect of IL-18 promotes both Th1 and Th2 responses, depending on the cytokine environment [54]. Allergen exposure and localized infection induce IL-18 expression. In humans as well as in mice, IL-18 is involved in the pathogenesis of *S. aureus*-associated AD [55,56]. Previous investigators have reported that staphylococcal enterotoxin A (SEA) results in an increase in IL-18 expression in vitro and in vivo [57,58]. Zedan et al. reported that the serum IL-12 and IL-18 concentration is associated with AD severity [59].

There has also been considerable interest in the role of Th17 and Th22 cells in the immunopathogenesis of AD [60,61]. To date, the Th17 cell pathway has been extensively investigated in various chronic inflammatory diseases and has been found to contribute to the onset of acute AD, although the role of Th17 cells in AD is relatively small in comparison to their role in psoriasis [62]. Koga et al. [63] demonstrated a strong association between acute AD severity and circulating Th17 cells. Th22 cells, IL-22-producing helper T cells, were different from the Th1, Th2, and Th17 cells. With AD, the Th22 cells function differently according to the age of the patients and their AD severity. Infants with AD show only a Th2/Th1 cell imbalance, whereas adults with AD exhibit Th22/Tc22 cell subsets [64]. Additionally, Th22 cells lead to the production of high levels of TNF-α and IL-13 [65,66,67]. The number of Th17 cells and the level of IL-17 expression were decreased in AD patients with severe symptoms, whereas the Th2 and Th22 cell subsets showed a positive association with AD severity [68]. A current phase II trial evaluates the efficacy and safety of anti-IL22 monoclonal antibody in treating patients with AD [69].

In summary, high quantities of allergens, including staphylococcal toxins, were transported through a damaged skin barrier to induce degranulation of MCs and the release of inflammatory mediators during the acute phase response of AD. Damaged epithelial cells trigger TSLP release that further promotes a Th2-type response in the skin. Although subsets of Th1, Th2, Th17, and Th22 cells co-exist in the acute AD response, the Th2 subtype is mainly involved in the acute phase of AD. Responses by the Th1, Th2, and Th22 subsets contribute to the chronic phase of AD.

## 3. Pathogenesis of AD from a Non-Immunologic Point of View

The mechanism of AD pathogenesis has been outlined in two differing hypotheses. The first proposes that the primary defect resides in an immunologic disturbance that leads to IgE-mediated sensitization, with epithelial-barrier dysfunction regarded as an outcome of local inflammation. The second proposes that an intrinsic defect in the epithelial cells results in the barrier dysfunction and the immunologic effects are considered a secondary symptom [1].

The skin barrier offers dual protective functions. As an inside–outside barrier, it guards against water loss, and, in the other direction, the outside–inside barrier prevents the entry of harmful substances from the environment including irritants, allergens, and microorganisms [70]. The stratum corneum of the skin acts as the permeability barrier and it consists of a lipid-enriched intercellular space and protein-rich cells (corneocytes).

A decrease in skin barrier function may be caused by the downregulation of the cornified envelope genes (loricrin and filaggrin), reduced levels of ceramide, abnormal keratin differentiation, increased levels of endogenous proteolytic enzymes, and an enhanced loss of transepidermal water (TEWL) [71,72]. Non-lesional AD skin exhibits a defect in the permeability barrier function [70]. Furthermore, the decreased barrier function of the skin triggers allergen sensitization and predisposes such AD patients to the development of food and respiratory allergies [22].

Excessive use of detergents, shampoos, and soaps can impair the barrier function of the skin and irritate the skin. Similarly, house dust mite allergens can contribute as enzymes that directly impair the permeability barrier of the skin, as well as immediately causing hypersensitivity reactions [73,74]. Reducing the temperature and humidity can reduce the number of house dust mites [75]. Furthermore, skin irritation and chronic eczema can be produced by prolonged low exposure to irritants in living areas [76].

Filaggrin aggregates keratin filaments into compact bundles and modifies the granular cell layer and the composition of keratinocytes [77]. Filaggrin interacts with lamellar bodies and reduces the availability of filaggrin metabolites, leading to changes in skin surface pH and skin hydration [78]. Null mutations within the *FLG* gene encoding filaggrin have been identified in approximately 30% of AD patients [79,80,81]. Additional studies suggest that *FLG* mutations lead to the early onset of AD and the development of asthma [82]. In a recent animal study, filaggrin-deficient mice developed spontaneous AD-like skin inflammation independent of the adaptive immune response, whereas adaptive immunity was necessary for the progression of impaired lung function [83]. Moreover, there was a significant relationship between AD with the *FLG* mutation and the peanut allergy mediated by IgE, indicating increased skin permeability and the consequent enhanced exposure to allergens [84]. A few genetic studies have demonstrated a linkage between polymorphisms in *SPINK5* or the stratum corneum chymotryptic enzyme and AD [85], although this has not been confirmed by other studies [86,87,88,89]. Netherton Syndrome is caused by mutations in *SPINK5* encoding the serine protease inhibitor known as the lympho-epithelial Kazal-type-related inhibitor (LEKTI), and this has specific clinical parallels to AD patients.

The reduction in barrier proteins is predicted to result from the downregulation of genes encoding for skin barrier proteins including filaggrin [82,90] and from the upregulation of Th2-type cytokines levels [91,92]. Significant associations between epidermal barrier defects and Th2 polarization in AD patients with filaggrin gene mutations can be partially explained by the enhanced penetration of allergens through the damaged epidermis [93,94]. Th2-type cytokines are involved in the pathogenesis of AD by decreasing the expression of skin barrier proteins including filaggrin [95,96,97], resulting in the increased penetration of pathogens and allergens.

Tight junctions are another extensively studied component of the skin barrier [98]. These are formed by a complex of transmembrane and intracellular proteins found in simple and stratified mammalian epithelia. In 2002, Tsukita and Furuse showed that claudin 1 deficiency in mice led to high TEWL and liver abnormalities, culminating in death [99]. The lesional skin of AD patients contains significantly decreased claudin 1 expression, but no claudin 4 reduction, when compared to the skin of non-atopic individuals [100,101,102]. Reduced claudin 1 appears to be related to an increased risk of infection by herpes simplex virus type 1 in individuals with AD [103]. There is also an inverse correlation between the expression of claudin 1 and the presence of the immune response markers of Th2 [100].

## 4. The Effect of *S. aureus* and Its Toxins on AD

There is a large group of microorganisms that colonize the skin; rather than passive inhabitants, they actively interact with host cells and influence the innate immune response [104]. The human skin microbiome is composed of Firmicutes (genus *Staphylococcus*), Actinobacteria (genus *Corynebacterium* and *Propionibacterium*), Bacteroidetes, and Proteobacteria [105,106]. A child’s skin microbiome positively influences early-life immune development away from allergic over- sensitization [107]. There is poor bacterial diversity in active AD lesions, with a predominance of *S. aureus*; once the patient has regained control over their AD, their bacterial milieu is then at least partially recovered [98]. In one study, treatment with emollient creams for 84 days improved clinical symptoms in 72% of children with AD, whose skin microbial diversity was restored to that characterized on non-lesional skin [108]. Yet, one recent study has found that 12-month-old infants with AD had not been colonized with *S. aureus* before they developed AD [109]. Randomized clinical trials have assessed the therapeutic effects of probiotics for AD treatment. However, the efficacy of probiotics for treating AD has not yet been demonstrated, especially when compared to traditional treatment modalities [110,111,112,113,114,115]. However, Zipperer et al. showed that colonization by the nasal commensal bacterium *S. lugdunensis* producing a novel cyclic peptide antibiotic lugdunin, which significantly reduced *S. aureus* carriage rate in humans. It suggests that lugdunin or the commensal bacteria may be an important source for the development or discovery of new antibiotics [116].

AD can be triggered or exacerbated by scratching/irritants, chemicals, allergens, and toxins such as staphylococcal enterotoxins (SEs) [57,117]. Host–microbe interactions at the skin surface play an important role in the immunopathogenesis of AD. Some AD patients may possess IgE sensitization against microbial antigens expressed by *Candida albicans*, *Malassezia**,*** or *S. aureus* [118,119,120]. In one study, TEWL was significantly higher among *S. aureus*-positive patients when compared with *S. aureus*-negative patients with AD [118]. Further, the increase in TEWL was proportional to the increase in bacterial load, and an increased TEWL was observed in patients who were sensitized to all three skin-associated microorganisms (*Candida*, *Malassezia* and *S. aureus*) compared to patients who were sensitized to none, one, or two of them [118]. Levels of IgE antibodies against *Malassezia* have been found to be higher in AD patients than in healthy controls. In addition, oral itraconazole or ketoconazole significantly improves the severity of clinical symptoms in AD patients after 1–2 months of daily treatment [119]. Additionally, monocyte-derived DCs are generated from *M. furfur* in peripheral blood, inducing significant production of IL-1β, IL-18, and TNF-α [121]. Another microbe implicated in the exacerbation of AD includes *S. epidermidis*, which may also be more abundant in AD patients [122]. Interestingly, the number of these commensal bacteria increases during exacerbations of AD, which is suggestive of a compensatory mechanism for the control of *S. aureus* [123].

**Figure 2:**
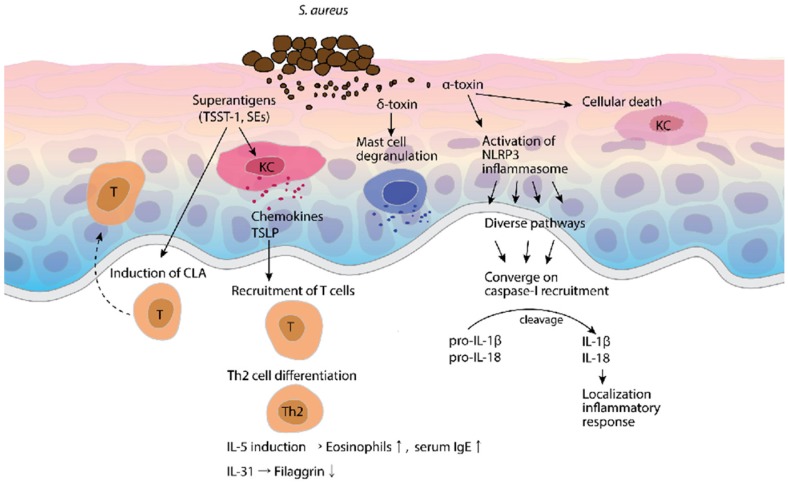
Mechanisms of *S. aureus* and its toxins on atopic dermatitis (AD). *S. aureus* and its toxins provide several mechanisms that result in AD. The *S. aureus* superantigens have the ability to induce cutaneous lymphocyte-associated antigen (CLA) expression as a skin-homing receptor on circulating T cells. Keratinocyte-derived chemokines and thymic stromal lymphopoietin (TSLP) induce the recruitment of T cells, Th2 cell differentiation, and the induction of T cells to secrete IL-5 and IL-31. The δ-toxin is an inducer of mast cell (MC) degranulation [125] and the α-toxin activates the nucleotide-binding oligomerization domain receptor protein 3 (NLRP3) inflammasome that eventually results in caspase-1 recruitment, and thereby leads to localized inflammatory responses via IL-1β and IL-18 secretion [126].

*Staphylococci* can produce many forms of infection both through their capacity to multiply and spread widely in tissues and through their production of many extracellular substances. Some of these substances are enzymes, and while others are considered toxins, they may function as enzymes. *S. aureus* produces hemolysins (α-toxin, δ-toxin), Panton-Valentine leukocidin, exfoliative toxins, enterotoxins, and superantigens (toxic shock syndrome toxin-1, staphylococcal enterotoxin B). In addition to the role of *S. aureus* in innate immunity, staphylococcal products including peptidoglycan, α-toxin, lipoteichoic acid, and superantigens activate cells, result in AD pathogenesis (Figure 2) [124].

The family of staphylococcal superantigens includes the toxic-shock syndrome toxin-1, SEs, and SE-like toxins [127,128,129,130,131]. The superantigens upregulate the expression of the cutaneous lymphocyte-associated antigen (CLA) as a skin-homing receptor on the surface of circulating T cells and the release of keratinocyte-derived chemokines that recruit circulating T cells. In addition, superantigens selectively induce the differentiation of T cells into Th2 cells, secreting a pruritogenic cytokine, IL-31, that controls filaggrin expression [132]. Superantigens downregulate IL-17-dependent induction of the AMP in keratinocytes by inhibiting the production of IL-17 and IL-22 from Th17 cells [133]. Furthermore, AD patients frequently demonstrate increased levels of IgE and production of IgE specific for these superantigens, whose levels are positively correlated with AD severity [134].

Staphylococcal bacteria commonly express at least four cytolytic toxins that appear to have roles in both host damage/inflammation and virulence. Recent studies have shown that δ-toxin is potent in stimulating MC degranulation. Although IgE is not necessary for δ-toxin-mediated MC degranulation, the presence of IgE enhances δ-toxin-induced MC degranulation in the absence of antigen [125]. Pore-forming staphylococcal α-toxin is a destructive cytolytic toxin that directly acts on cell membranes by binding to sphingomyelin molecules leading to α-toxin-induced keratinocyte cell death and Th2 cytokine production [135,136]. Brauweiler et al. provided evidence that Th2 cytokine-exposed keratinocytes can be sensitive to α-toxin-induced cell death [39]. Hong et al. demonstrated that α-toxin, particularly of the extracellular vesicle-associated form, induced both AD-like skin inflammation and skin barrier disruption, and suggested that extracellular vesicle-associated α-toxin could be used as a new diagnostic and therapeutic method for the regulation of AD [137].

Filaggrin deficiency results in both increased antigen penetration into the skin and enhanced viral and bacterial growth in the skin, as well as increased susceptibility to the cytotoxic effects of staphylococcal α-toxin [138,139,140]. SEs constitute a family of streptococcal and staphylococcal exotoxins with homologous sequences that share a similar function. These toxins are produced by enterotoxigenic strains, mainly *S. aureus* [141].

Niebuhr et al. [61] showed that IL-22 expression was strongly induced by staphylococcal α-toxin and staphylococcal enterotoxin B (SEB), both in freshly isolated peripheral blood memory T cells and in Th22 cells derived from memory T cells in long-term cell culture without polarization. The same authors demonstrated in another study that the staphylococcal α-toxin and SEB were strong inducers of IL-22 secretion in CD4^+^ T cells and that the sublytic concentrations of α-toxin and SEB were strong inducers of IL-22 secretion in peripheral blood mononuclear cells (PBMCs) [61].

## 5. Toxins That Inhibit AD and Their Inhibitory Mechanisms

AD treatment using natural materials or toxins has been assessed in many studies (Table 1). While some studies on natural materials for AD treatment have been published, toxin-involved AD treatment has rarely been examined in the literature. There are several recognized AD models, yet none of these truly reflect the actual pathophysiology of this human disease [142]. Moreover, studies that have focused on the therapeutic effects are limited, since most of the results only relate to AD alleviation in animal models via Th2 responses. Therefore, studies identifying the therapeutic effects of toxins in AD treatment are scarce.

Inhibition of Th2 responses has been proven to be the underlying molecular mechanism for the treatment of AD with both natural materials and toxins [15,143,144,145,146]. However, the application of these compounds to treat chronic AD induced by the Th1/Th22 response to improve the skin barrier and to remove *S. aureus* along with other microbial antigens has not been widely studied.

Products of soybean fermentation are popular food sources in Asian countries, and isoflavones, including genistein and daidzein, are the major component. They are reported to have antioxidant and anti-inflammatory effects [147]. Yeh et al. [143] found that feeding AD mice with a fermented legume product led to the attenuation of cutaneous Th2 responses, as evidenced by a decreased epidermal thickness, lower levels of CXCL11, IL-5, and IL-13 expression, and less eosinophil infiltration when compared to controls in BALB/c mice. Genistein also suppresses the development of AD-like skin lesions in NC/Nga mice [148].

Bee venom has long been used in Korea, China, and Japan as a traditional medicine. It contains apamin, melittin, adolapin, phospholipase A_2_, and an MC-degranulating peptide [150]. Recent studies have demonstrated that bee venom application induced a significant anti-inflammatory response via the inhibition of inflammatory mediators, similar to what is achieved by treatment with non-steroidal anti-inflammatory drugs [151,152,153]. Further, Han et al. [154] have shown that bee venom treatment has anti-inflammatory effects in the skin and a rapid cicatrizing effect on wounds in rats. Kim et al. reported that the anti-itch effect of bee venom ameliorated compound 48/80-induced AD symptoms by inhibiting MC degranulation [15]. Lee et al. [155] demonstrated that bee venom and its component, melittin, mediated the anti-inflammatory effect via nuclear factor (NF)-κB signaling, confirming that activation of the p38 pathway was important in the activation of IL-1β and TNF-α during inflammatory reactions.

*Polygonum tinctorium* (Naju Jjok) was reported to suppress the total clinical severity in 2,4-dinitrofluorobenzene-induced AD-like skin lesions in NC/Nga mice [144]. *Polygonum tinctorium* significantly suppressed the levels of IL-4 and IgE in the serum of 2,4-dinitrofluorobenzene-induced AD mice [144]. Tryptanthrin is a natural product from *Polygonum tinctorium* and it is known to have anti-pyretic, anti-inflammatory and detoxicant actions in traditional Korean medicine [156,157]. Tryptanthrin inhibited TSLP expression via blocking caspase-1 activity in MCs in an AD murine model [158].

Ginseng and ginsenosides have frequently been used for the treatment of chronic inflammatory diseases. Kim et al. [145] showed that oral administration of ginseng extract markedly improved *Dermatophagoides farinae* (house dust mite) extract-induced AD-like symptoms in NC/Nga mice. Cultivated ginseng suppressed the development of AD-like symptoms by controlling the Th1 and Th2 responses in the skin lesions of mice, and thymus- and activation-regulated chemokine expression by blocking TNF-α/IFN-γ-induced NF-κB activation in HaCaT cells [159]. Cho et al. [149] demonstrated that oral administration of red ginseng could inhibit the development of AD-like skin lesions in NC/Nga mice both systemically and locally by inhibiting DCs, TSLP, and the Th2 response.

To screen potential herbs, Yun et al. conducted a systematic review of in vivo studies of AD-like skin models. Among 22 cited studies, 21 herbs have been reported to reduce AD-like skin lesions in mouse models by suppressing the Th2 cell response [146]. Regarding the herbal treatment of AD, it is crucial to monitor possible side effects including sensitization and contact allergies from the herbal substances. In a Cochrane review that included 28 randomized controlled trials, one study reported a single severe adverse effect, and 24 studies revealed minor adverse events, including transiently elevated liver enzymes, which were resolved immediately after stopping Chinese herbal medicine treatment [160].

## 6. Inflammasome Expression and Function in AD

The inflammasome, which is responsible for the activation of inflammatory processes, has been shown to induce cell pyroptosis. Cell pyroptosis is an inflammatory form of programmed cell death that is different from apoptosis. Our understanding of innate mmunity has been advanced following the identification of three interacting families of pathogen sensors: RIG-I-like receptors (RLRs), nucleotide-binding oligomerization domain (NOD)-like receptors (NLRs), and TLRs. Since the year 2000, several active studies on inflammasomes and inflammatory diseases have revealed the mechanisms behind inflammatory responses. Cytoplasmic pattern recognition receptors sense microbial metabolites, effectors, nucleic acids, and other danger signals to form a multiprotein complex called the inflammasomes [161]. Following the activation of the inflammasome sensor, these diverse pathways converge on the recruitment of caspase-1 to activate the inflammasome and caspase-1 autoproteolysis. Activated caspase-1 cleaves pro-IL-18 and pro-IL-1β into the active secreted forms [126].

Most toxins activating the inflammasomes are pore-forming toxins that activate the nucleotide-binding oligomerization domain receptor protein 3 (NLRP3) inflammasome [126]. Bacterial pore-forming toxins induce the loss of cellular potassium and subsequent indirect NLRP3 activation. The study of inflammasomes that mediate pore formation began with the analysis of marine and fungal ionophores and is now expanding beyond bacterial toxins [162]. Mold pore-forming mycotoxins [163], viral viraporins [164], melittin and the small cationic pore-forming peptide found in bee venom are single membrane-spanning alpha helical proteins [165]. The frog *Bombina maxima* is known to express non-bacterial pore-forming toxins. When the NLRP3 inflammasome is activated by a signal from a microorganism or a crystal, IL-1β is released and local inflammatory reactions are induced. In contrast, the sustained activation of the NLRP3 inflammasome triggers multi-organ involvement and periodic fever in patients with inflammatory diseases [166].

AD is an inflammatory skin disorder, and its etiology and complex pathophysiology cannot be fully explained by using skin transplants or mouse models. The majority of AD patients’ skin is colonized by *S. aureus* [167], and hemolysins and bacterial lipoproteins from *S. aureus* induce the activation of the NLRP3 inflammasome [168,169]. In addition, *Malassezia* yeasts can induce the activation of the NLRP3 inflammasome in antigen-presenting cells through Syk-kinase signaling [170]. Dai et al. [171] demonstrated that mite-allergen-mediated activation of NLRP3 inflammation and the subsequent release of the IL-1 family proteins were important for AD development. Douglas et al. [172] provided evidence that inflammasome activation was the important pathogenic process in the initiation of skin disease in a chronic proliferative dermatitis mouse model. Single nucleotide polymorphisms in NOD1 and NOD2 that induce loss-of-function mutations are associated with AD development [19,173,174,175]. It has been reported that the AD severity index is inversely correlated with the expression of the NLR family pyrin domain containing 1 (NALP1) protein [18]. Niebuhr et al. [176] demonstrated that AD lesional skin exhibited a reduced expression of caspase-1 and NLRP3, and that caspase-1-dependent IL-1β secretion by staphylococcal α-toxin stimulation was impaired in mononuclear cells of AD patients when compared with healthy controls. Schuepbach-Mallepell et al. [17] also showed that inflammasome activation inhibited the upregulation of TSLP mRNA. Overall, these results indicate an inhibitory effect by the inflammasome on AD development in humans.

## 7. Conclusions

Our discussion explores the relationship between toxins and AD. Even though numerous studies are in progress, the mechanism of toxins in inflammasomes and the possibility of toxins being used as a treatment option for AD still need to be studied. AD is a complex genetic disease characterized by allergen sensitization derived from the interaction between immunologic mechanisms and an impaired skin barrier. As part of this process, physical irritation, chemical allergens, and toxins are involved. Although a precise explanation for this process has not yet been fully elucidated, a great deal has been revealed through research from various angles. Many studies on the effects of staphylococcal toxins on the progress of AD are currently being undertaken. In addition, studies regarding the administration of antiseptics to AD patients are continually being undertaken, even though this is largely controversial. Further studies into the inhibitory mechanisms of AD and the alleviating role of toxins are therefore needed.

## Figures and Tables

**Table 1:** Natural materials and toxins that have inhibitory effects on AD and their related mechanisms.

Toxin/Natural Material	Results and Mechanism	References
*Saccharomyces cerevisiae* legume fermented product	Skin: thickness ↓, eosinophil ↓, IL-5 ↓, IL-13 ↓, CXCL11 ↓ Lymph node: IL-4 ↓, IL-17A ↓	[143]
Bee venom	Skin: scratching ↓, mast cell degranulation ↓ TNF-α ↓, IL-1β ↓	[15]
*Polygonum tinctorium* (Naju Jjok)	Skin: thickness ↓, inflammatory cells ↓, TSLP ↓ Serum: IL-4 ↓, IgE ↓	[144]
Ginseng extract	Skin: IL-4 ↓, IL-5 ↓, IL-13 ↓, IFN-γ ↓, TNF-α ↓, CCL17 ↓ Serum: IgE ↓, CCL17↓	[145]
Korean red ginseng extract	Skin: thickness ↓, water loss ↓, inflammatory cells ↓ TNF- α ↓, TSLP ↓, Serum: IgE ↓	[149]
Herbs	See reference Suppression of Th2 response	[146]
